# Supplementary material for: Hybrid Models and Biological Model Reduction with PyDSTool
Source: PLoS Comput Biol. 2012 Aug 9;8(8):e1002628. doi: 10.1371/journal.pcbi.1002628 (PMC3415397; doi:10.1371/journal.pcbi.1002628)
Supplement: Text S4 — Complete source code for the PyDSTool package (version 0.88.120504). Includes API documentation and help files linking to web pages. This file is identical to the current public release on Sourceforge.net. (ZIP) [file pcbi.1002628.s004.zip › PyDSTool/html/PyDSTool.Toolbox.optimizers.tests.test_quadratic-pysrc.html]

xml version="1.0" encoding="ascii"?


PyDSTool.Toolbox.optimizers.tests.test\_quadratic


| Home | Trees | Indices | Help | PyDSTool |
| --- | --- | --- | --- | --- |

Package PyDSTool ::
Package Toolbox ::
Package optimizers ::
Package tests ::
Module test\_quadratic

[hide private]

[frames] | no frames]

# Source Code for Module PyDSTool.Toolbox.optimizers.tests.test\_quadratic

```
 1  #!/usr/bin/env python
 
 2  
 
 3  # Matthieu Brucher
 
 4  # Last Change : 2007-08-24 11:20
 
 5  
 
 6  """
 
 7  Class defining a quadratic function
 
 8  """ 
 9  
 
10  import numpy 
11  from numpy.testing import * 
12  set_package_path() 
13  from optimizers import criterion, step, optimizer, line_search 
14  restore_path() 
15  
 


16 -class Quadratic:


17    """
 
18    A simple quadratic function
 
19    """ 


20 -  def __call__(self, x):


21      """
 
22      Get the value of the quadratic function at a specific point
 
23      """ 
24      return (x[0] + 2* x[1] - 7)**2 + (2 * x[0] + x[1] - 5)**2

25  
 


26 -  def gradient(self, x):


27      """
 
28      Evaluates the gradient of the function
 
29      """ 
30      return numpy.array([2 * (x[0] + 2* x[1] - 7) + 4 * (2 * x[0] + x[1] - 5), 4 * (x[0] + 2* x[1] - 7) + 2 * (2 * x[0] + x[1] - 5)], dtype = numpy.float)

31  
 


32 -  def hessian(self, x):


33      """
 
34      Evaluates the gradient of the function
 
35      """ 
36      return numpy.array([[10, 8], [8, 10]], dtype = numpy.float)

37  
 


38 -class test_Quadratic(NumpyTestCase):


39    """
 
40    Global test class with a quadratic function
 
41    """ 


42 -  def check_simple_gradient(self):


43      startPoint = numpy.zeros(2, numpy.float) 
44      optimi = optimizer.StandardOptimizer(function = Quadratic(), step = step.GradientStep(), criterion = criterion.RelativeValueCriterion(0.00001), x0 = startPoint, line_search = line_search.SimpleLineSearch(alpha_step = 0.001)) 
45      assert_almost_equal(optimi.optimize(), numpy.array([1, 3], dtype = numpy.float))

46  
 


47 -  def check_simple_newton(self):


48      startPoint = numpy.zeros(2, numpy.float) 
49      optimi = optimizer.StandardOptimizer(function = Quadratic(), step = step.NewtonStep(), criterion = criterion.RelativeValueCriterion(0.00001), x0 = startPoint, line_search = line_search.SimpleLineSearch()) 
50      assert_almost_equal(optimi.optimize(), numpy.array([1, 3], dtype = numpy.float))

51  
 


52 -  def check_damped_gradient_relative(self):


53      startPoint = numpy.zeros(2, numpy.float) 
54      optimi = optimizer.StandardOptimizer(function = Quadratic(), step = step.GradientStep(), criterion = criterion.RelativeValueCriterion(0.00001), x0 = startPoint, line_search = line_search.DampedLineSearch(damped_error = 0.001, min_alpha_step = 0.0001)) 
55      assert_almost_equal(optimi.optimize(), numpy.array([1, 3], dtype = numpy.float))

56  
 


57 -  def check_swp_frgradient_relative(self):


58      startPoint = numpy.zeros(2, numpy.float) 
59      optimi = optimizer.StandardOptimizer(function = Quadratic(), step = step.FRConjugateGradientStep(), criterion = criterion.criterion(ftol=0.000001, iterations_max=1000, gtol = 0.0001), x0 = startPoint, line_search = line_search.StrongWolfePowellRule()) 
60      assert_almost_equal(optimi.optimize(), numpy.array([1, 3], dtype = numpy.float))

61  
 


62 -  def check_swp_cwgradient_relative(self):


63      startPoint = numpy.zeros(2, numpy.float) 
64      optimi = optimizer.StandardOptimizer(function = Quadratic(), step = step.CWConjugateGradientStep(), criterion = criterion.criterion(ftol=0.0000001, iterations_max=1000, gtol = 0.0001), x0 = startPoint, line_search = line_search.StrongWolfePowellRule()) 
65      assert_almost_equal(optimi.optimize(), numpy.array([1, 3], dtype = numpy.float), decimal=4)

66  
 


67 -  def check_swp_prpgradient_relative(self):


68      startPoint = numpy.zeros(2, numpy.float) 
69      optimi = optimizer.StandardOptimizer(function = Quadratic(), step = step.PRPConjugateGradientStep(), criterion = criterion.criterion(ftol=0.000001, iterations_max=1000, gtol = 0.0001), x0 = startPoint, line_search = line_search.StrongWolfePowellRule()) 
70      assert_almost_equal(optimi.optimize(), numpy.array([1, 3], dtype = numpy.float))

71  
 


72 -  def check_swp_dpgradient_relative(self):


73      startPoint = numpy.zeros(2, numpy.float) 
74      optimi = optimizer.StandardOptimizer(function = Quadratic(), step = step.DConjugateGradientStep(), criterion = criterion.criterion(ftol=0.000001, iterations_max=1000, gtol = 0.0001), x0 = startPoint, line_search = line_search.StrongWolfePowellRule()) 
75      assert_almost_equal(optimi.optimize(), numpy.array([1, 3], dtype = numpy.float))

76  
 


77 -  def check_swp_dypgradient_relative(self):


78      startPoint = numpy.zeros(2, numpy.float) 
79      optimi = optimizer.StandardOptimizer(function = Quadratic(), step = step.DYConjugateGradientStep(), criterion = criterion.criterion(ftol=0.000001, iterations_max=1000, gtol = 0.0001), x0 = startPoint, line_search = line_search.StrongWolfePowellRule()) 
80      assert_almost_equal(optimi.optimize(), numpy.array([1, 3], dtype = numpy.float))

81  
 


82 -  def check_simple_marquardt(self):


83      startPoint = numpy.zeros(2, numpy.float) 
84      optimi = optimizer.StandardOptimizer(function = Quadratic(), step = step.MarquardtStep(), criterion = criterion.criterion(gtol=0.00001, iterations_max=200), x0 = startPoint, line_search = line_search.BacktrackingSearch()) 
85      opt = optimi.optimize() 
86      assert_almost_equal(optimi.optimize(), numpy.array([1, 3], dtype = numpy.float), decimal = 5)

87  
 
88  if __name__ == "__main__": 
89    NumpyTest().run() 
90
```

  


| Home | Trees | Indices | Help | PyDSTool |
| --- | --- | --- | --- | --- |

|  |  |
| --- | --- |
| Generated by Epydoc 3.0.1 on Fri May 4 15:24:15 2012 | http://epydoc.sourceforge.net |
